# Pseudo-right atrial earliest activation suggesting a functionally inferred left-sided slow conduction zone in adenosine-sensitive atrial tachycardia

**DOI:** 10.1016/j.hrcr.2026.07.010

**Published:** 2026-07-18

**Authors:** Toshihiko Gokan, Yuya Nakamura, Morio Ono, Junko Honye, Toshiro Shinke

**Affiliations:** 1 Division of Cardiology, Department of Medicine, Showa Medical University Hospital, Tokyo, Japan; 2 Department of Cardiology, Kikuna Memorial Hospital, Yokohama, Japan

**Keywords:** Adenosine-sensitive atrial tachycardia, Slow conduction zone, Entrainment pacing, High-density mapping, Para-Hisian region

**Key Teaching Points**

• In para-Hisian adenosine-sensitive atrial tachycardia, a fragmented and spatially dispersed right atrial earliest activation pattern, resembling a “splattered-milk-like” appearance, may represent passive breakthrough from a left-sided substrate rather than the true arrhythmogenic substrate.
• Stepwise biatrial directional capture mapping, using orthodromic and antidromic capture responses from right atrial, coronary sinus, and left atrial pacing sites, can help functionally infer the location and orientation of a left-sided slow conduction zone that is not directly visualized on activation mapping.
• When orthodromic capture is absent from the high posteroseptal right atrial region but present from the distal coronary sinus and left atrial appendage, transseptal left atrial mapping should be considered before empirical ablation within the non-coronary aortic sinus.

## Introduction

Para-Hisian adenosine-sensitive atrial tachycardia (AT) is a reentrant supraventricular tachycardia that reproducibly terminates with low-dose adenosine or adenosine 5′-triphosphate (ATP), with the earliest atrial activation site (EAAS) often recorded near the His bundle.^1^^,^^2^ Multidirectional capture mapping, as proposed by Yamabe et al,^3^^,^^4^ uses orthodromic and antidromic capture of the EAAS from different pacing sites to infer the presence and orientation of a functional slow conduction zone (SCZ) that is not directly visualized on local activation time (LAT) maps. We report a case in which the apparent para-Hisian right atrial (RA) EAAS was most consistent with passive breakthrough rather than the true substrate, thereby creating a false right-sided target. Stepwise biatrial capture mapping supported a functionally inferred left-sided SCZ and redirected ablation strategy away from empirical ablation within the non-coronary aortic sinus toward a transseptal left atrial (LA) approach.

## Case report

A 66-year-old man without structural heart disease presented with recurrent palpitations and exertional dyspnea. Sinus rhythm electrocardiography and transthoracic echocardiography were unremarkable. Clinical episodes showed a long-RP narrow-QRS supraventricular tachycardia that reproducibly slowed and terminated with intravenous ATP 1 mg, with ventriculoatrial (VA) block and no change in PR interval (Figure 1A).

**Figure 1:**
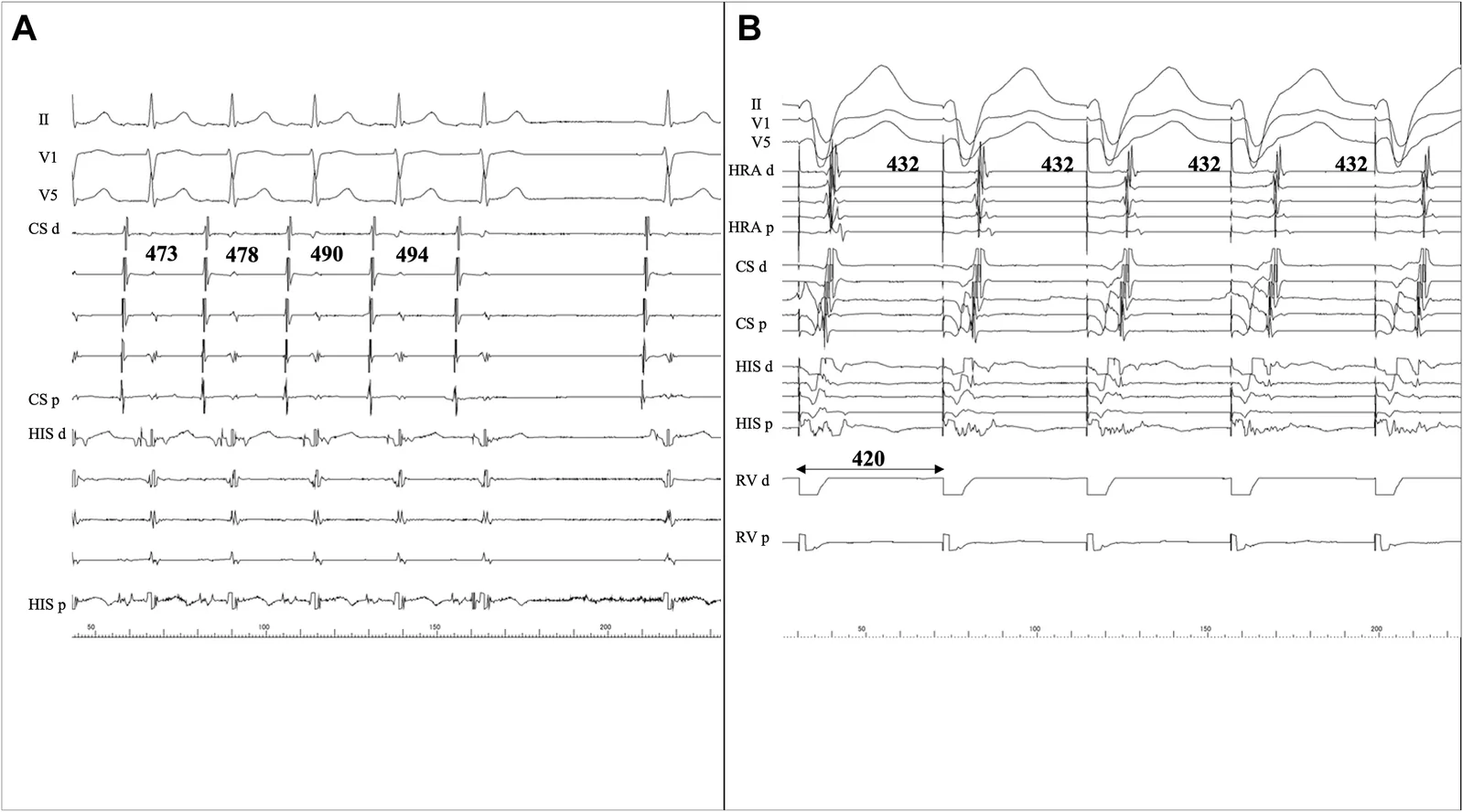
Intracardiac electrograms during adenosine-sensitive atrial tachycardia. **A:** A low dose (1 mg) of adenosine 5′-triphosphate, used as an adenosine agent, was administered as a rapid intravenous bolus. This resulted in gradual prolongation of the tachycardia cycle length, followed by termination of the tachycardia, without prolongation of the atrial–His interval. These findings were consistent with adenosine-sensitive atrial tachycardia rather than atrioventricular nodal reentrant tachycardia (AVNRT) or orthodromic reciprocating tachycardia (ORT). **B:** Intracardiac electrograms recorded during ventricular entrainment pacing during the tachycardia demonstrated ventriculoatrial (VA) dissociation, confirming that the tachycardia was not dependent on retrograde VA conduction and excluding ORT as the mechanism, while making AVNRT unlikely, prompting right atrial activation mapping.

During the electrophysiology study, multipolar catheters were positioned in the high RA, His bundle region, right ventricular apex, and coronary sinus (CS). Baseline Atrial–His and His–ventricle intervals were within normal limits, and the clinical AT was reproducibly induced by high RA burst pacing. Ventricular entrainment during tachycardia demonstrated VA dissociation (Figure 1B). RA LAT mapping with an Advisor HD Grid catheter and the EnSite X system identified a para-Hisian EAAS (Figure 2). The earliest region was fragmented and spatially indistinct, with multiple discontinuous early breakthrough islands rather than a compact focal source, raising suspicion of passive RA breakthrough from a functionally inferred left-sided substrate. However, the mechanistic interpretation primarily relied on the subsequent biatrial directional capture responses. Capture direction was assessed at the EAAS, as described in previous entrainment studies.^3^^,^^4^ The last entrained atrial electrogram and the first postpacing atrial electrogram at the EAAS were designated A1 and A2, respectively. Orthodromic capture was considered present when the EAAS was activated with a long conduction time and an electrogram morphology similar to that during tachycardia, with the A1–A2 interval consistent with the tachycardia cycle length. Antidromic capture was considered present when the EAAS was directly captured by the paced wavefront, with a different local activation sequence or electrogram morphology. Because the tachycardia cycle length fluctuated mildly, overdrive pacing was performed at a pacing cycle length 10–20 ms shorter than the cycle length at that time; the figures indicate these intervals as pacing cycle length. Entrainment pacing from multiple RA sites resulted in uniform antidromic capture of the EAAS, so entrainment and pacing were performed from the distal CS, which produced orthodromic capture of the EAAS (Figure 3). The last entrainment sequence showed an atrial–His–ventricular (A2-H-V) pattern,^5^ and the maximal change in VA interval (ΔVA) across atrial pacing sites was 47 ms.^6^ These findings were diagnostic of AT.^5^^,^^6^ Therefore, distal CS was considered to be located upstream of the SCZ in the AT circuit, prompting transseptal LA mapping. Because pacing from the clinically termed high posteroseptal RA region, anatomically adjacent to the posterosuperior interatrial fold, failed to produce orthodromic capture of the EAAS, an SCZ directed toward the non-coronary aortic sinus was considered less likely, and transseptal LA mapping was prioritized over empirical ablation within the non-coronary aortic sinus.

**Figure 2:**
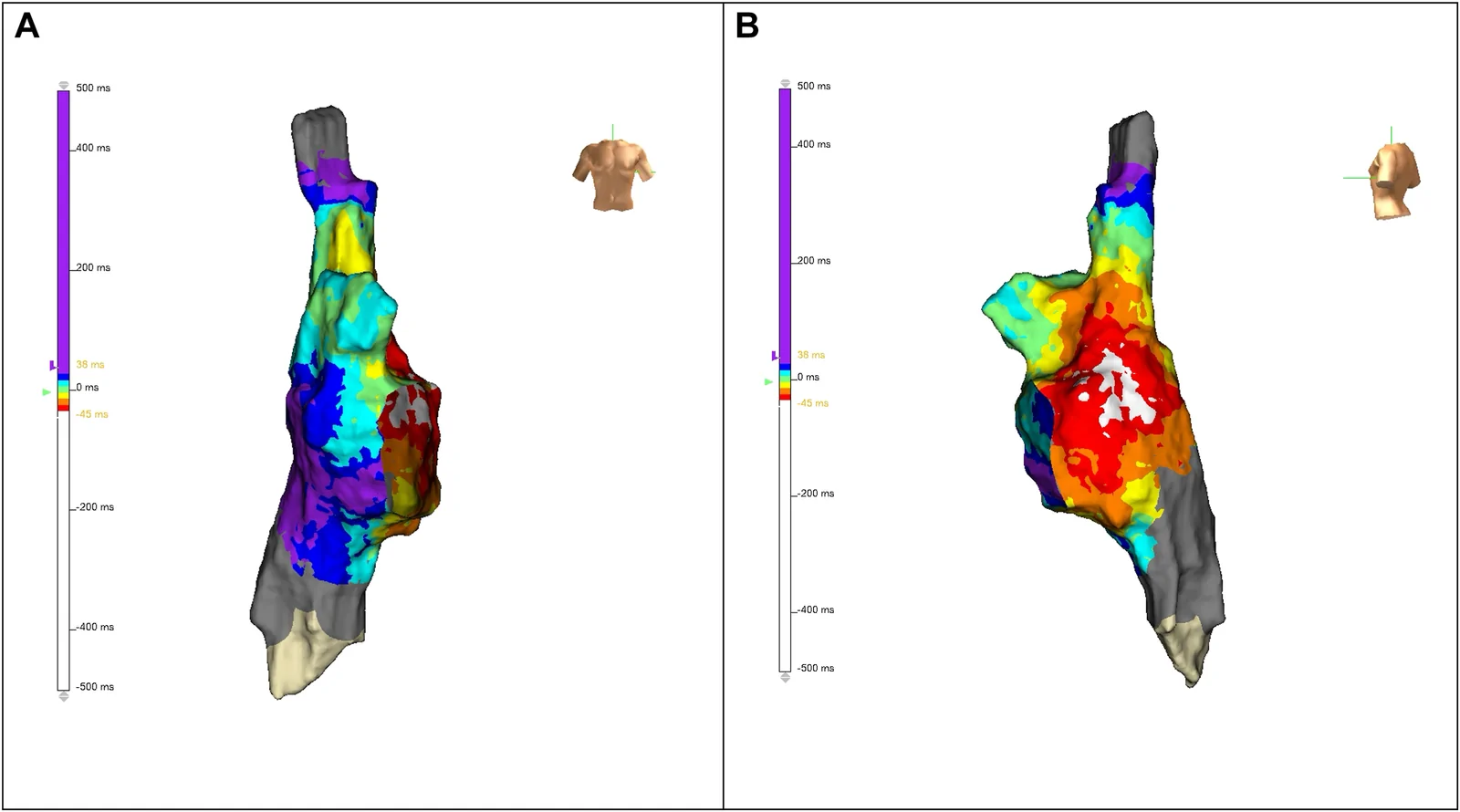
Right atrial (RA) high-density activation mapping during adenosine-sensitive atrial tachycardia. **A:** Anteroposterior view. **B:** Septal view of a RA local activation time map during the clinical tachycardia, created using the EnSite X EP System (Abbott) and an Advisor HD Grid Mapping Catheter (Abbott). The apparent earliest atrial activation site (EAAS) was located in the para-Hisian region, and the overall activation appeared to spread centrifugally from this region; however, the EAAS was fragmented and spatially dispersed, with multiple discontinuous early islands rather than a single discrete focal source, a pattern suggestive of passive RA breakthrough from a functionally inferred left-sided circuit (a “splattered-milk-like” appearance).

**Figure 3:**
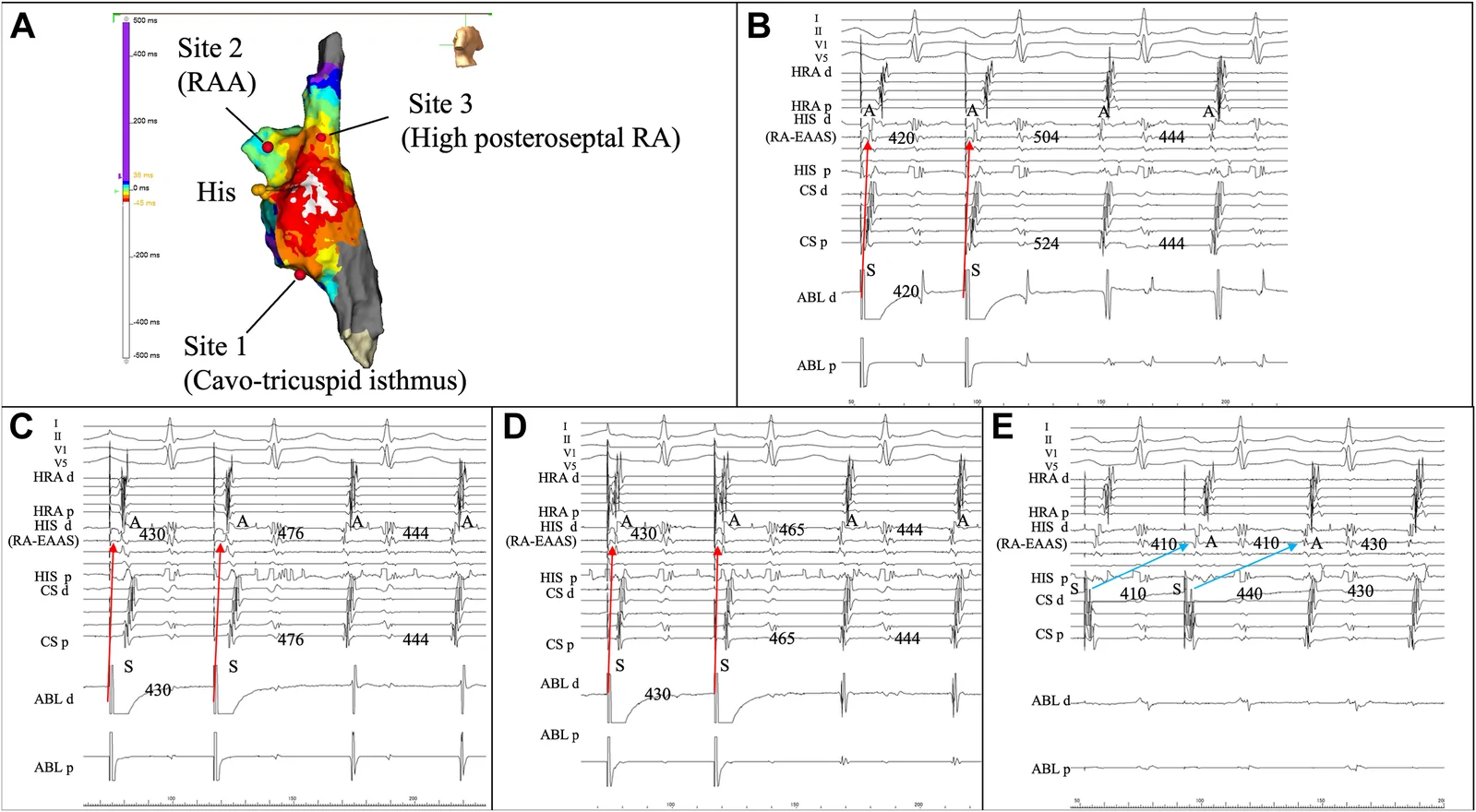
Right atrial (RA) activation mapping and multi-directional capture mapping. **A:** An RA local activation time (LAT) map during the tachycardia. **B:** Cavo-tricuspid isthmus (Site 1) entrainment pacing captured the earliest atrial activation site (EAAS) antidromically, indicating a downstream site. **C:** RA appendage (Site 2) pacing also captured the EAAS antidromically. **D:** Pacing from the clinically termed high posteroseptal RA region, anatomically adjacent to the posterosuperior interatrial fold (Site 3), also captured the EAAS antidromically. **E:** Distal coronary sinus pacing produced orthodromic capture of the EAAS.

After transseptal puncture, LA LAT mapping demonstrated a discrete EAAS in the infero-anterior LA vestibular region near the 10 o’clock position of the left atrioventricular junction, from which activation propagated centrifugally through working myocardium (Figure 4 and Supplemental Video 1). This LA EAAS preceded the para-Hisian RA earliest site by 11 ms. To localize the functional SCZ, entrainment and pacing were performed from several LA sites. The Advisor HD Grid catheter was positioned to surround the LA EAAS, allowing stable entrainment and pacing assessment around the apparent EAAS. Pacing from the septum, roof, and posterior wall resulted in antidromic capture of the LA EAAS, whereas pacing from the distal and basal LA appendage (LAA) produced orthodromic capture (Figures 5 and 6). These orthodromic responses supported the interpretation that the SCZ was functionally inferred to lie between the LAA region and the LA EAAS along the anterior LA near the basal LAA (schematically summarized in Figure 7A). As the pacing site was advanced stepwise from the distal LAA toward the LA EAAS using a comparable overdrive pacing cycle length, the capture pattern transitioned from orthodromic to antidromic capture, and tachycardia reproducibly terminated when the LA EAAS was antidromically captured.

**Figure 4:**
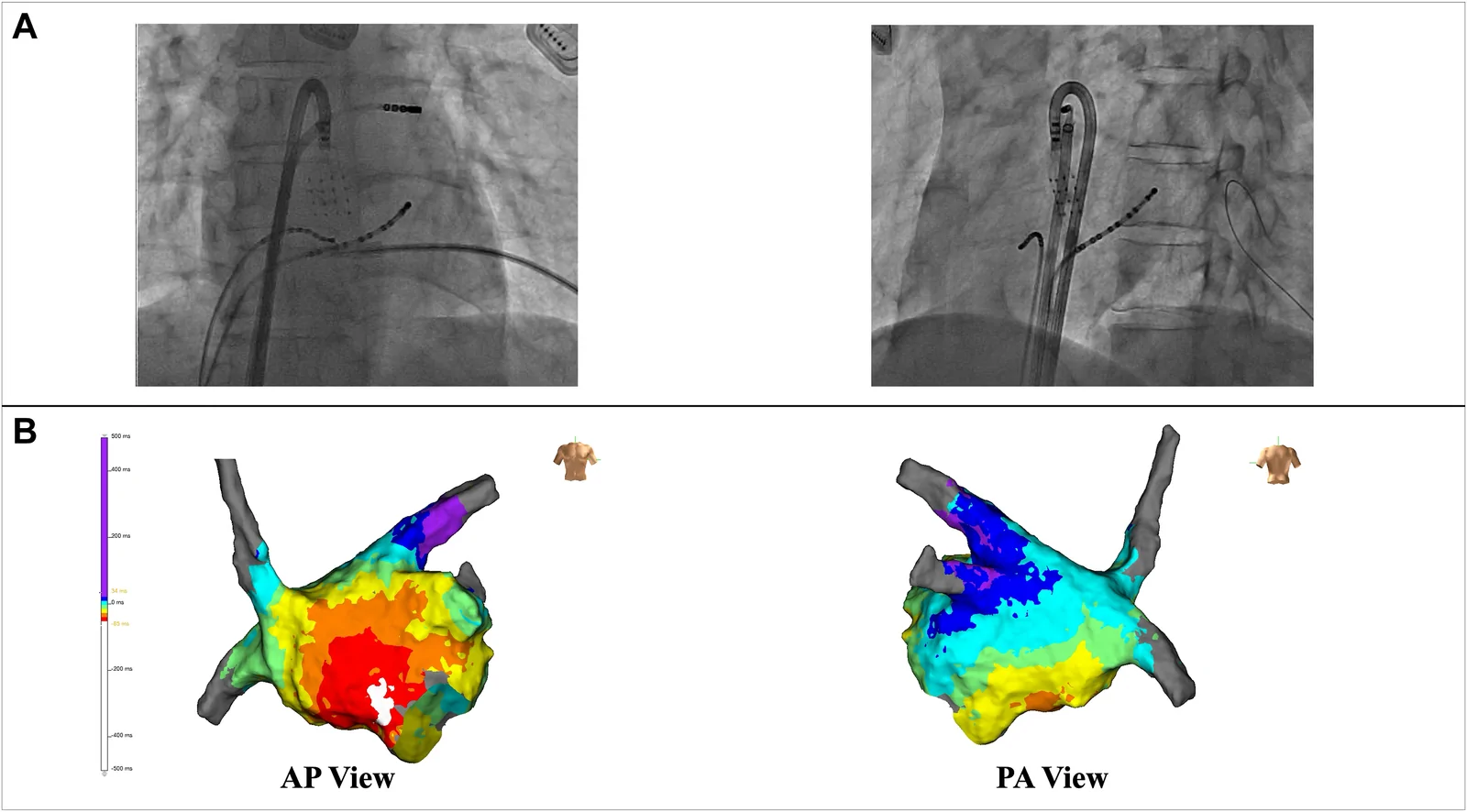
Identification of the left atrial (LA) earliest atrial activation site. Following transseptal puncture, LA mapping identified the earliest atrial activation site (EAAS) in the infero-anterior LA vestibular region near the 10 o’clock position of the left atrioventricular junction, preceding the para-Hisian right atrial (RA) EAAS by 11 ms. **A:** Fluoroscopic images in anteroposterior and left anterior oblique projections showing an ablation catheter and an Advisor HD Grid Mapping Catheter, placed at the LA EAAS, positioned in the infero-anterior wall of the LA. **B:** LA local activation time map during atrial tachycardia demonstrating centrifugal activation spreading in a concentric pattern from the LA EAAS, which was more clearly delineated than on RA mapping.

**Figure 5:**
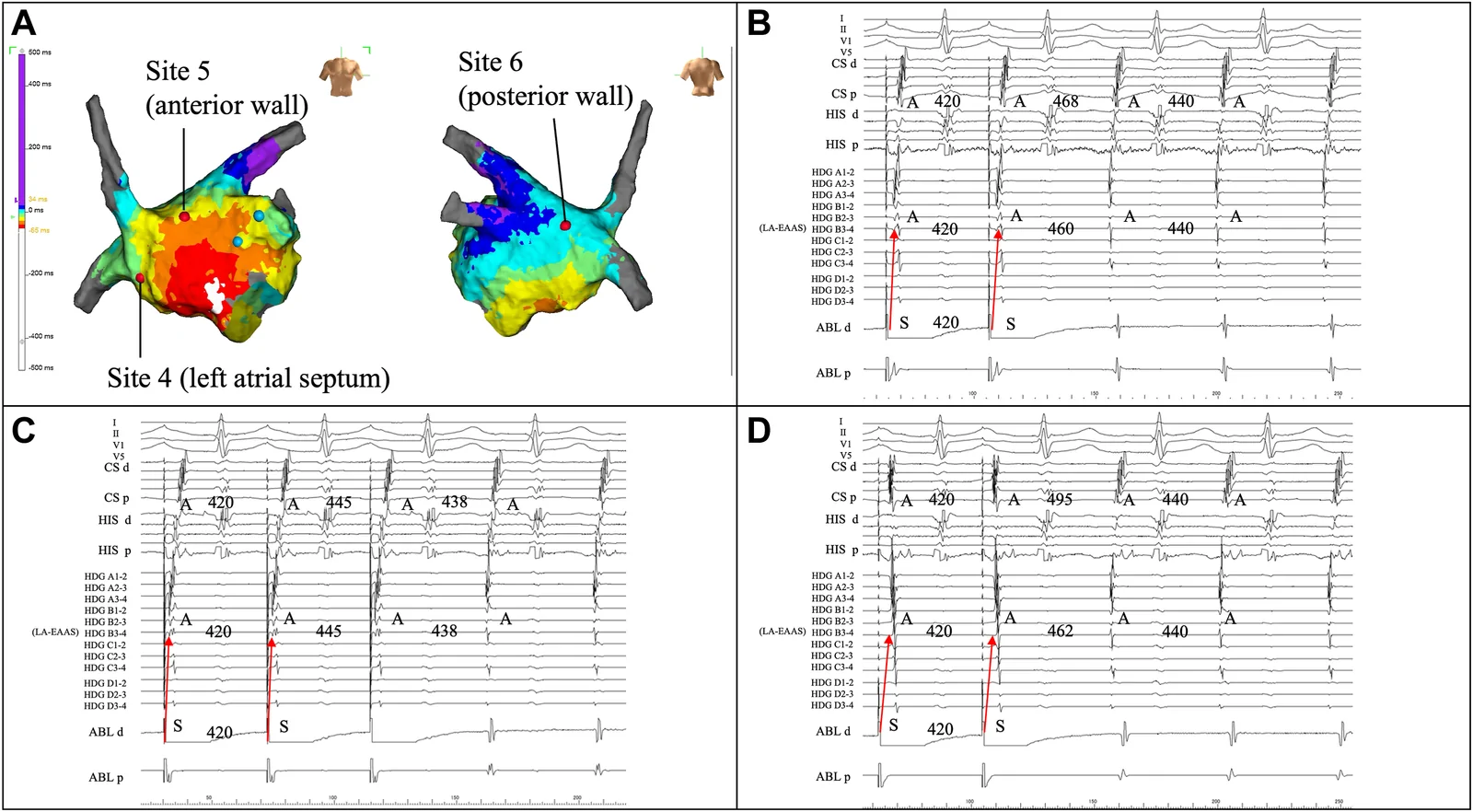
Left atrial (LA) activation mapping and multi-directional pacing**. A:** LA local activation time map during atrial tachycardia. *Red points* indicate sites from which the LA earliest atrial activation site (EAAS) was captured antidromically, whereas *blue points* indicate sites from which the LA EAAS was captured orthodromically. **B:** Intracardiac electrograms during entrainment pacing from Site 4 (LA septum), demonstrating antidromic capture of the LA EAAS. **C:** Intracardiac electrograms during entrainment pacing from Site 5 (supero-anterior wall), demonstrating antidromic capture of the LA EAAS**. D:** Intracardiac electrograms during entrainment pacing from Site 6 (posterior wall), demonstrating antidromic capture of the LA EAAS.

**Figure 6:**
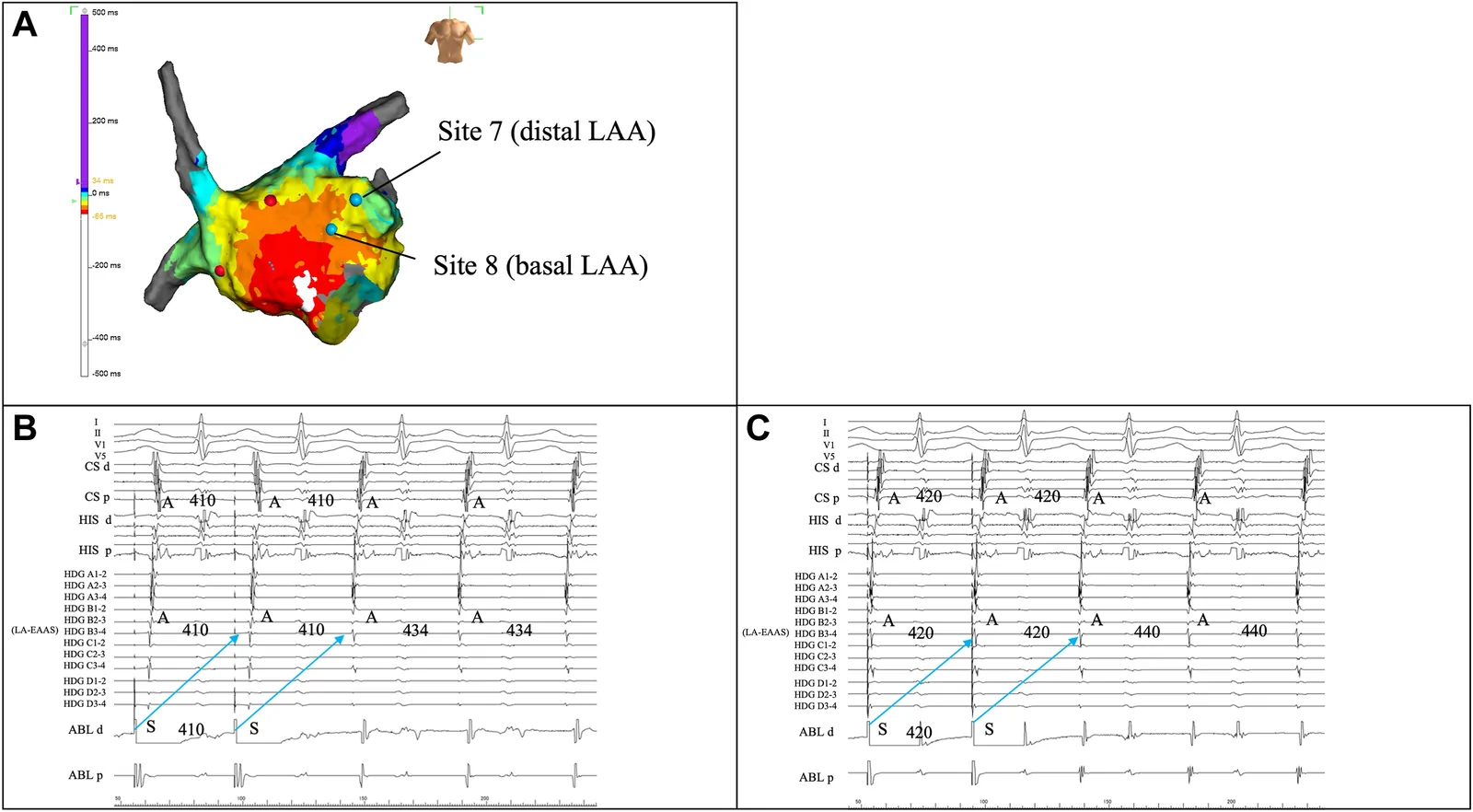
Orthodromic capture from the left atrial appendage (LAA). **A:** Left atrial (LA) local activation time (LAT) map during atrial tachycardia. *Red points* indicate sites from which the LA earliest atrial activation site (EAAS) was captured antidromically, whereas *blue points* indicate sites from which the LA EAAS was captured orthodromically. **B:** Intracardiac electrograms during overdrive pacing from Site 7 (distal LAA), demonstrating orthodromic capture of the LA EAAS. **C:** Intracardiac electrograms during overdrive pacing from Site 8 (basal LAA), demonstrating orthodromic capture of the LA EAAS.

**Figure 7:**
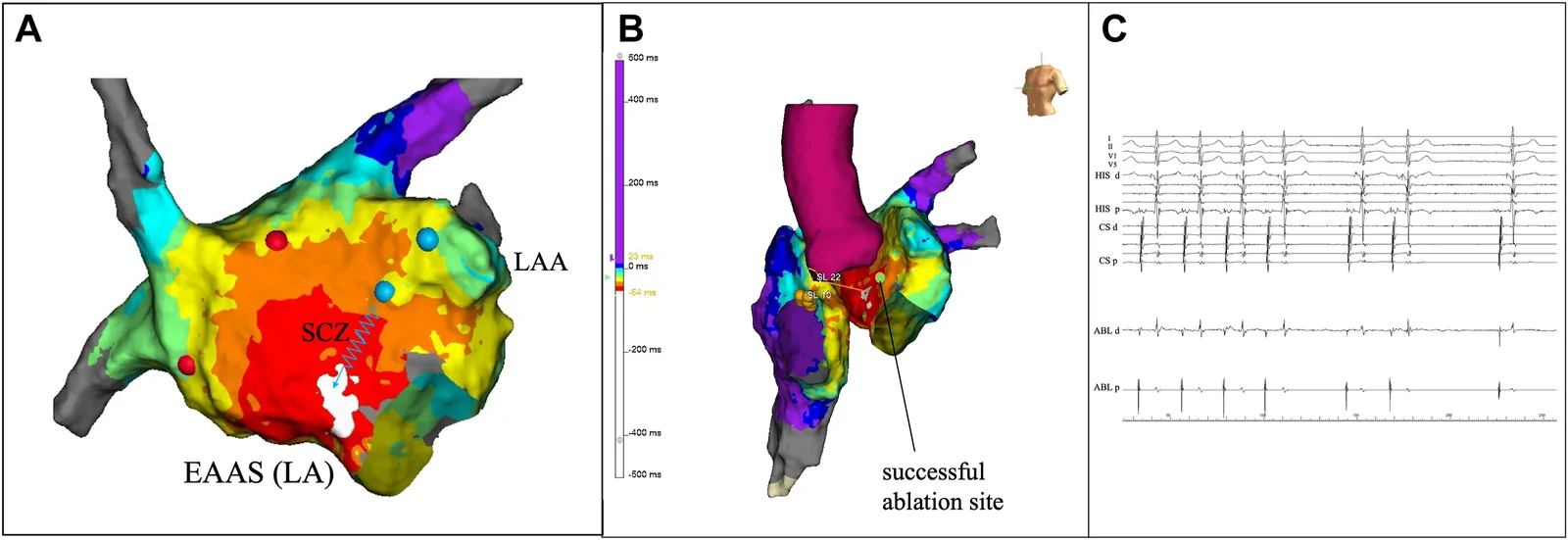
Proposed mechanism and computed tomography (CT)-fused biatrial mapping of the functionally inferred left-sided slow conduction zone. **A:** Left atrial (LA) local activation time (LAT) map showing centrifugal activation spreading in a concentric pattern from the LA earliest atrial activation site (EAAS). Based on orthodromic capture from the distal and basal left atrial appendage (LAA), the slow conduction zone was considered to lie closer to the EAAS than these LAA pacing sites. **B:** A CT-fused biatrial local activation time map with the aortic root. *Yellow points* indicate the right-sided His bundle recording site. The green marker denotes the successful ablation site. The distance from the non-coronary aortic sinus to the LA earliest atrial activation site (EAAS) was approximately 22 mm, whereas the distance from the non-coronary aortic sinus to the His bundle recording site was 10 mm. **C:** Intracardiac electrograms at the successful ablation site showing progressive slowing of the tachycardia followed by termination during radiofrequency energy delivery.

Biatrial activation maps were fused with computed tomography (CT), allowing direct comparison of the spatial relationships among the non-coronary aortic sinus, the His bundle, and the successful LA ablation site (Figure 7B). The distance from the non-coronary aortic sinus to the His bundle recording site (10 mm) was shorter than the distance from the non-coronary aortic sinus to the LA EAAS (22 mm), suggesting that empirical ablation within the non-coronary aortic sinus would not have been anatomically aligned with the inferred LA substrate in this case. These anatomic relationships supported prioritizing targeted transseptal ablation of the inferred LA SCZ as a more anatomically and mechanistically appropriate strategy. In contrast, the inferred LA target within the functional SCZ was anatomically remote from the His bundle. Radiofrequency energy at 30 W delivered at this inferred SCZ site led to gradual tachycardia slowing and termination 3 seconds after energy onset (Figure 7C). The AT became noninducible, and the PR interval, AH interval, His–ventricle interval, and Wenckebach rate remained unchanged. The patient was discharged the following day and remained free from recurrent AT without antiarrhythmic drugs during 1-year follow-up.

## Discussion

The principal diagnostic observation in this case is that the apparent para-Hisian RA earliest activation site behaved as a pseudo-breakthrough rather than the true substrate. A prior activation-mapping study showed that passive activation from adjacent tissue can create a pseudo-focal centrifugal pattern.^7^ In the present case, the fragmented and spatially dispersed RA earliest region was consistent with such a pseudo-focal breakthrough pattern. Uniform antidromic capture during entrainment pacing from multiple RA sites, followed by orthodromic capture during pacing from the distal CS and then the LAA, supported the interpretation that a functionally inferred left-sided SCZ was present. As pacing advanced stepwise from the LAA toward the LA earliest activation site, the transition from orthodromic to antidromic capture, together with tachycardia termination near that site, supported the interpretation that the LA earliest activation site was functionally linked to the circuit and likely represented the exit or an immediate downstream breakout.

The anatomical substrate underlying this observation should be interpreted with caution. The SCZ was not directly visualized but was functionally inferred from the directional capture responses, activation sequence, and successful ablation site. Because the entire circuit of adenosine-sensitive para-Hisian AT is generally not delineated by activation mapping alone, circuit size and microreentry or macroreentry classification could not be determined. Nonetheless, ATP sensitivity, established diagnostic criteria, directional capture responses, and termination at the inferred SCZ supported a reentrant mechanism. In the present case, the relevant region was most consistent with the LA vestibular region near the left atrioventricular junction, with possible involvement of the anterior interatrial groove. This anatomical interpretation may explain why the initial activation pattern appeared to arise from the para-Hisian RA. The anterior interatrial region contains myocardial continuities between the atrial chambers, including Bachmann’s bundle, and the atrial vestibules are also in myocardial contiguity in this region.^8^^,^^9^ Therefore, activation emerging from an LA vestibular or anterior interatrial groove region could produce fragmented breakthrough into the para-Hisian RA, creating the misleading impression of a right-sided substrate.

Prior reports from our group have described left-sided adenosine-sensitive AT and atypical left-sided SCZ orientation.^10^^,^^11^ In contrast, the present case emphasizes that the initial para-Hisian RA earliest activation pattern itself may be misleading, representing passive breakthrough that can create a false right-sided or non-coronary aortic sinus target. The absence of orthodromic capture from the clinically termed high posteroseptal RA region, together with orthodromic capture from the distal CS and LAA region, supported a left-sided SCZ and made empirical ablation within the non-coronary aortic sinus less appropriate. CT-integrated mapping strengthened this procedural implication because the non-coronary aortic sinus was closer to the His bundle than to the successful LA ablation site, whereas the transseptal LA approach allowed targeted ablation at the inferred SCZ remote from the His bundle.

### Limitations

This report has several limitations. First, it describes a single case. Second, the SCZ and putative circuit exit were inferred from directional pacing responses and activation timing rather than directly visualized. Third, systematic postpacing interval mapping in the LA was not performed because the tachycardia cycle length fluctuated, making its interpretation less reliable. Fourth, ablation within the non-coronary aortic sinus was not performed in this patient; therefore, the relative efficacy of that approach remains inferential. Nevertheless, the reproducible biatrial capture responses, CT-integrated spatial relationships, and successful transseptal ablation support, but do not prove, the proposed mechanism.

## Conclusion

In para-Hisian adenosine-sensitive AT, an apparent RA earliest activation site may represent pseudo-EAAS reflecting passive breakthrough from a functionally inferred left-sided substrate rather than the true substrate. Stepwise biatrial capture mapping may functionally localize a left-sided SCZ, support that the LA EAAS is linked to the circuit as the exit or an immediate downstream breakout, and help avoid empirical ablation within the non-coronary aortic sinus when that approach is not supported by pacing responses.

## Disclosures

The authors have no conflicts to disclose.

## Appendix Supplementary Data

Supplementary Video

Supplemetary Video 1
