# Supplementary material for: Pseudo-right atrial earliest activation suggesting a functionally inferred left-sided slow conduction zone in adenosine-sensitive atrial tachycardia
Source: HeartRhythm Case Rep. 2026 Jul 18;12(10):1351–7. doi: 10.1016/j.hrcr.2026.07.010 (PMC13687967; doi:10.1016/j.hrcr.2026.07.010)
Supplement: Supplementary file 1 [file mmc1.docx]

**Video Legend**

**Video 1. Biatrial activation map overlay during the adenosine-sensitive atrial tachycardia**

Biatrial three-dimensional local activation time maps with computed tomography fusion of the aortic root and a dynamic SparkleMap overlay are displayed during atrial tachycardia. Yellow points indicate the His bundle recording site. The maps demonstrate that the anterior left atrial wall is the earliest site of atrial activation in both atria. The non-coronary aortic sinus is closer to the His bundle recording site than to the left atrial earliest atrial activation site and the successful ablation site, supporting ablation at the left atrial slow conduction zone rather than empirical ablation within the non-coronary aortic sinus.
